# IoT Enabled Intelligent Sensor Node for Smart City: Pedestrian Counting and Ambient Monitoring

**DOI:** 10.3390/s19153374

**Published:** 2019-08-01

**Authors:** Fowzia Akhter, Sam Khadivizand, Hasin Reza Siddiquei, Md Eshrat E. Alahi, Subhas Mukhopadhyay

**Affiliations:** 1 Department of Engineering, Macquarie University, Sydney, NSW 2109, Australia; 2 Department of Electrical and Electronic Engineering, Uttara University, Dhaka 1230, Bangladesh; 3 Shenzhen Institute of Advanced Technology, Chinese Academy of Sciences, Shenzhen 518055, China

**Keywords:** intelligent sensor, the direction of travel, ambient monitoring, smart city

## Abstract

An Internet of Things (IoT) enabled intelligent sensor node has been designed and developed for smart city applications. The fabricated sensor nodes count the number of pedestrians, their direction of travel along with some ambient parameters. The Field of View (FoV) of Fresnel lens of commercially available passive infrared (PIR) sensors has been specially tuned to monitor the movements of only humans and no other domestic animals such as dogs, cats etc. The ambient parameters include temperature, humidity, pressure, Carbon di Oxide (CO_2_) and total volatile organic component (TVOC). The monitored data are uploaded to the Internet server through the Long Range Wide Area Network (LoRaWAN) communication system. An intelligent algorithm has been developed to achieve an accuracy of 95% for the pedestrian count. There are a total of 74 sensor nodes that have been installed around Macquarie University and continued working for the last six months.

## 1. Introduction

With the proliferation of technological advancement, the idea of a smart city is emerging day by day. A city is named as smart once its intelligent and sustainable infrastructure is integrated with advanced technological solutions [1]. The essential purpose of building a smart city is providing a sustainable, environment-friendly life to its residents. It is predicted that 66% of the world population is expected to live in urban areas by 2050 [2]. Due to the industrial revolution and rapid urbanization, human societies are in great strain on resource consumption and environmental issues [3]. Thus, it has become an important issue to keep track of footpath traffic and environmental parameters for urban planning, retail development, major event crowd assessments, pedestrian safety, traffic flow management and assessments of the street development.

Researchers are seeking interest in people counting in office buildings as well as public places such as train stations. There are numerous researches that have been carried out on human counting using different technologies in the indoor environment [4,5,6]. Occupancy counting in smart buildings is implemented via video cameras, thermal cameras, sensors, radio frequency identification (RFID) and Wi-Fi [7] probe requests [8,9,10,11].

One of the most extensively used methods of human counting is the usage of a video camera. It also has a wide range of applications in the surveillance of potential areas for detecting unusual events, tracking customers in retail stores to control and monitor the movements of assets, monitoring elderly and sick people staying at home alone, recognize and track people [12]. The number of people in each frame of the video camera are determined based on the image processing algorithm. Some methods count the number of people by counting heads, whereas some methods count faces from the captured images [13]. However, the system count and actual count vary due to the spatial characteristics of the area under surveillance (confined compared to open area), features extracted from the image, expected response time (real-time requirements compared to offline processing) and the maximum size of the crowd. Moreover, the performance of the video camera degrades due to environmental conditions such as sudden luminosity changes, camera location and angle of view. Furthermore, the process of counting people is complicated and very costly in terms of computing and money [14]. There is an infringement of privacy. This technique allows accuracy up to 95% for an indoor surveillance environmental and 85% for outdoor applications [15].

A thermal camera is another popular method for human detection. It is also used for vehicle detection, search for victims at night, spot smoldering fires inside a wall and detecting overheating electrical wiring. The thermal camera functions by detecting the infrared energy emitted by an object which is known as a heat signature and creating an image electronically based on information about the temperature differences. Compared to optical imaging, thermal imaging cameras are advantageous for night-time video surveillance. It can be used where pedestrians’ privacy respect cannot be violated. However, it also outperforms the bad results obtained from visible images due to occlusions or closing texture [16]. Moreover, the size and weight of the camera are quite big (4 kg to 7 kg), and it consumes a high current (250 mA), which requires additional circuitry to run by an IoT enabled microcontroller based system [17].

PIRs are other important technologies for human detection. These low-cost motion sensors respond when an infrared (IR) emitting subject (humans or animals) passes through its FoV [18]. PIR sensors have been employed by many researchers for human detection, localization and gait velocity estimation at indoor surveillance. Zappi et al. (2010) proposed an array of Passive Infrared sensors (PIR) placed on the ceiling to determine the number of people passing the hallway. They detected the position and direction of movement from the analogue output signal of PIR which consists of two peaks. However, the drawback of the system is that it cannot detect more than one person at a time [19]. Wang et al. (2014) showed two fusion methods for determining the building occupant level. The first method assumed independent observations of the multiple systems, whereas the second method exploits the correlation among the multiple information sources. They combined two methods and compared the experimental results with the results obtained from individual RFID or video cameras. The maximum accuracy of this fusion method is 73% only [20].

The usage of PIR sensors does not violate human privacy, unlike the video camera. It needs 3.3–5 V supply to drive the PIR sensors. It consumes only 3 mA current while sensing [21]. Therefore, it is possible to use the sensors in a microcontroller-based system. As the current consumption is deficient, there is no necessity to use any amplifier [22]. Therefore, the circuitry becomes very simple. These sensors have been widely used for human presence detection in the indoor environment [23]. However, it is not used in the outdoor environment to date as its performance degrades with the change in temperature and humidity.

The radio frequency identification (RFID) technology is also used for the identification of objects and people. There are two main components of an RFID network such as RFID readers and RFID tags. It then reflects signals with the tags’ unique identification number to the reader. The RFID reader emits electromagnetic waves and activates the RFID tags [24]. RFID tags are classified as active and passive. Between these two, passive RFID tags are vastly used due to long life and cost-effectiveness. Among various applications, some potential applications are occupancy counting in smart buildings, building evacuation during natural disasters or terrorist attack, bus and railway stations, smart parking systems, asset tracking, bill payment in toll gates, track prisoners, etc. [25,26,27].

The utilization of Wi-Fi probe requests is another important aspect of human counting and tracking inside office buildings. Ciftler et al. (2018) used Wi-Fi probe requests for human occupancy monitoring and tracking in smart buildings. Probe requests are continuously broadcasted signals from devices having Wi-Fi technology, such as smartphones, laptops and tablets. The unencrypted probe requests can be captured and decoded using passive sniffers without connecting to a particular network. The proposed method was able to differentiate the peak and off-peak hours of individual zones and track zone-level occupancy tracking with a maximum accuracy of 90%. This is adaptable for localization of pinch points of a building, tracking and occupancy counting for indoor surveillance [28].

In addition to the development of occupancy counting in smart buildings, some cities of the USA, Europe and Australia have already started adopting smart technologies in their cities for pedestrian counting and collecting environmental data. These data are collected to have an adequate understanding of the ongoing trends of pedestrian activity. This helps to plan and respond to emergencies. Moreover, this data helps the city planner to take a decision quickly. The pedestrian counting system of Melbourne uses both camera and laser-based sensors, whereas Auckland, New York, Dublin, and Barcelona use only camera-based technology. Some systems (Auckland, New York and Liverpool) are typically installed under canopies or on street poles. Some other systems (Melbourne, Dublin and Barcelona) need separate installation facilities, which are an extra financial burden. The data collected are transferred to the server and uploaded on the website for public use via the 3G communication box. Although the usage of 3G communication covers a more extended range compared to Wi-Fi, ZigBee [29] or other communication protocols, they consume excessive device energy [30]. Moreover, the power consumption of the camera-based system is higher compared to the sensor-based prototype. A summary of the existing pedestrian counting system is shown in Table 1. Although the existing systems can serve the purpose, power consumption, cost and people’s privacy concerns should also be addressed. Therefore, it has become an important issue to come up with a low-power, low-cost, compact, accurate system. Having additional features will be more advantageous.

After reviewing the technologies used for pedestrian counting, it is decided to use the PIR sensors in this research as it is a cost-effective, low-power sensor compared to other existing technologies. The drawbacks of the PIR sensors are considered while designing the prototype. Therefore, the developed prototype will be able to overcome the downsides of the sensors. This paper presents a novel compact system that not only provides the pedestrian count but also gives information about the pedestrian’s direction of travel and environmental data such as temperature, humidity, pressure, CO_2_ and TVOC. The novel contributions of this paper can be summarized as follows:

1. The sensitivity of the PIR sensors has been tuned, and a specific section from the Fresnel lens is chosen for receiving the IR emission from 1 m above the ground. Therefore, domestic animals such as dogs or cats will not be detected while crossing the footpath as the maximum shoulder height of a domestic dog is less than 100 cm [37].
2. A novel method of the pedestrian count and direction detection system has been designed. Three PIR sensors cover the detection region across the width of footpath in such a way that the detection regions of the sensors do not overlap. Each of the three sensors is horizontally paired with another three sensors such that each pair can validate their detection as well as find the direction of travel of the pedestrian. Therefore, the system can provide highly accurate results, up to 95% compared to manual counting.
3. A microcontroller-based low-power sensing device along with the low-power communication system has been proposed to increase the battery life. The system can be installed on the street pole without altering the other infrastructure of the city.

## 2. Materials and Methods

This section presents the necessary explanation for developing the smart counting system. It also explains the FoV of Fresnel lens of the PIR sensors, the novel method of pedestrian detection and finding the direction of travel, proposed algorithm, installation system, study locations, data transmission and collection system.

### 2.1. Field of View for PIR Sensor

**Figure 1:**
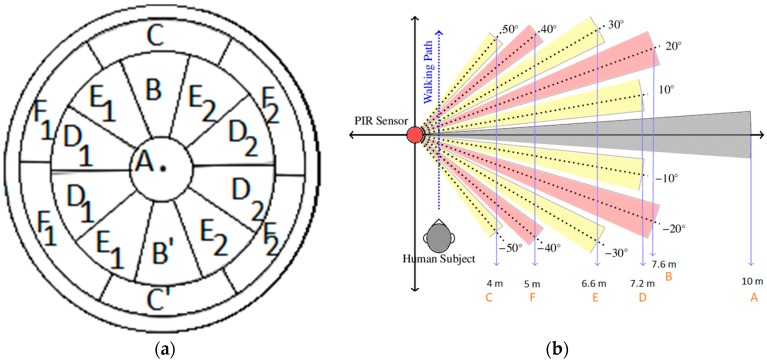

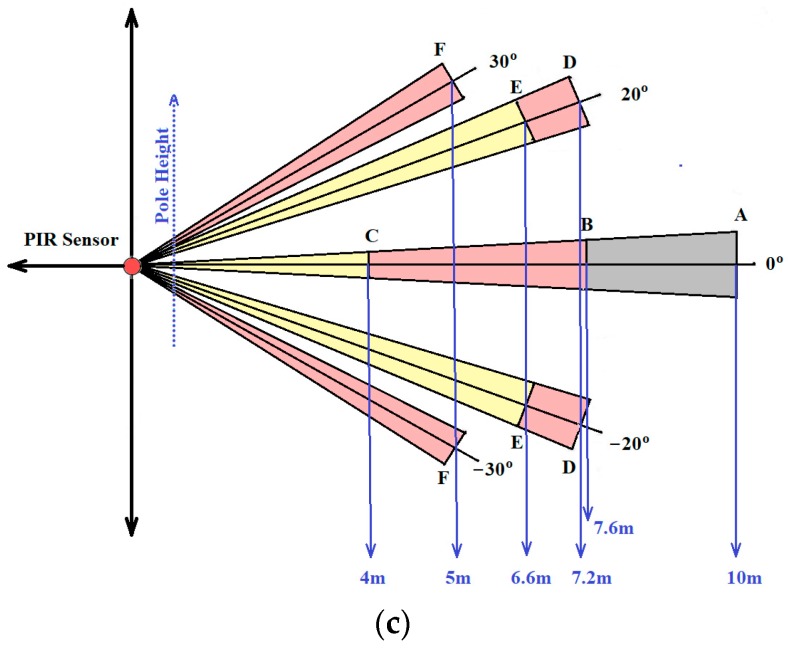
(**a**) Segmentation of Fresnel lens [21], (**b**) top View of FoV of Fresnel lens [18], and (**c**) side view of FoV of Fresnel lens.

A PIR sensor consists of two sensing elements and a Fresnel lens. The Fresnel lens focuses the emitted IR radiation to the sensing element. The sensing element absorbs the IR radiation and converts it into heat, which is converted into a minute electric current by the pyroelectric crystalline material [18]. A Fresnel lens is composed of a series of concentric grooves having a standard focal length. The significant advantage of the Fresnel lens is that the propagation direction of light does not change within a medium but only deviates at the surfaces of a medium. The different faceting and sub-lenses of the Fresnel offer a range of detection areas. The focal point of the FoV is divided into A-F sections (Figure 1a), which are 10 degrees apart from each other. The different sections (A–F) of the Fresnel lens offer a detection range from 4 m–10 m at full sensitivity, as shown in Figure 1b. The detection length can be decreased to 1.8 m at reduced sensitivity. The usage of the A-section at full sensitivity implies the installation of the sensor node at 11 m height of the pole. This is not possible in the practical scenario as the maximum height of the lamp posts is 6.5 m [38]. Therefore, it is necessary to operate the PIR sensors at a reduced sensitivity mode rather than full sensitivity. The detection length of each section of the Fresnel lens is measured by walking towards that section from a far distance covering other sections with Polyvinyl chloride (PVC) tape. Figure 1b,c shows the top view and side view of the FoV of the Fresnel lens of the PIR sensor (Parallax Inc., Rocklin, CA, USA, 555-28027 PIR sensor module) at full sensitivity while Table 2 shows the experimentally verified detection lengths of the A to F sections at full and reduced sensitivity.

### 2.2. Method for Pedestrian Detection

**Figure 2:**
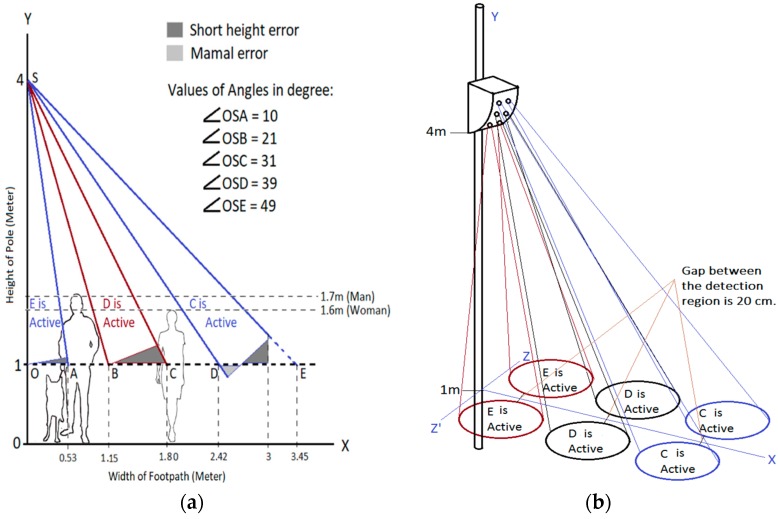
(**a**) Height of the system and counting mechanism, (**b**) 3-D view of the detection regions of the PIR sensors for the proposed system.

This research aims to cover 3 m wide footpath and limit the detection length to 1 m from the street level for avoiding the domestic animal. Three pairs of PIR sensors are used to develop the node for counting pedestrian and finding the direction of travel. The sensor node is mounted at 4 m of the electric pole, as shown in Figure 2a. The bottom PIR pair is placed at 0° from the y-plane so that a person walking adjacent to the pole can also be detected. Each section of the Fresnel lens has 10° FOV. Therefore, the detection region of the bottom pair can be calculated as follows:

(1) $$\mathrm{Opposite}=\mathrm{Adjacent}\times \tan\theta \Rightarrow \mathrm{OA}=\mathrm{OS}\times \tan∠OSA=3\;m\times \tan10^{0}=0.53\;m$$

To find the appropriate focus angle of the middle pair of the PIR sensors, it is assumed that a person is standing at the edge of the detection region of the bottom pair as shown in Figure 2a. The middle pair is placed in such a way that the person detected by the bottom pair does not fall in the coverage region of the middle pair. From the graphical representation of this scenario, it is determined that the middle pair detection region should start at 21° from the y-plane. Therefore, this middle pair detection region ends at 31° from the y-plane. The minimum and maximum coverage regions of the middle pair are calculated as follows:

(2) $$\mathrm{OB}=\mathrm{OS}\times \tan∠OSB=3\;m\times \tan(21^{0})=1.15\;m$$

(3) $$\mathrm{OC}=\mathrm{OS}\;\times \tan∠\mathrm{OSC}=3\;m\times \tan(21^{0}+10^{0})=1.80\;\mathrm{mv}$$

Detection regions of the top pair are calculated in a similar manner and values are shown in Figure 2a. Coverage regions of the sensors do not overlap due to applying this method while locating the PIR sensors. Therefore, the error can be minimized.

The required detection length of the bottom, middle and top PIR sensors can be found using the formula of a right-angle triangle, which is, $hypotenuse=\sqrt{{Adjacent}^{2}+{opposite}^{2}}.$ The values of SA, SB and SD are calculated as 3 m, 3.17 m and 3.87 m, respectively. The appropriate selection of the sections of the Fresnel lens is necessary to minimize the error. If the selected section detects more area under the 1 m height of clearance, then tall domestic pets, such as dogs will be detected. When the selected section detects less area above the 1 m clearance, then pedestrians having a shorter height such as 1.1–1.2 will not be detected. As it is not possible to fine-tune the detection length of the commercial PIR sensor to achieve a full area of coverage, the section should be selected that provides minimum error region. Therefore, E, D and C- sections are kept active for the bottom, middle and top pairs, respectively. Bottom and middle PIR sensor pairs are operated at reducing sensitivity, whereas the top pair operates at full sensitivity to minimize the error. People on wheel-chair also come under the detection region.

### 2.3. Development of the Sensor Node

**Figure 3:**
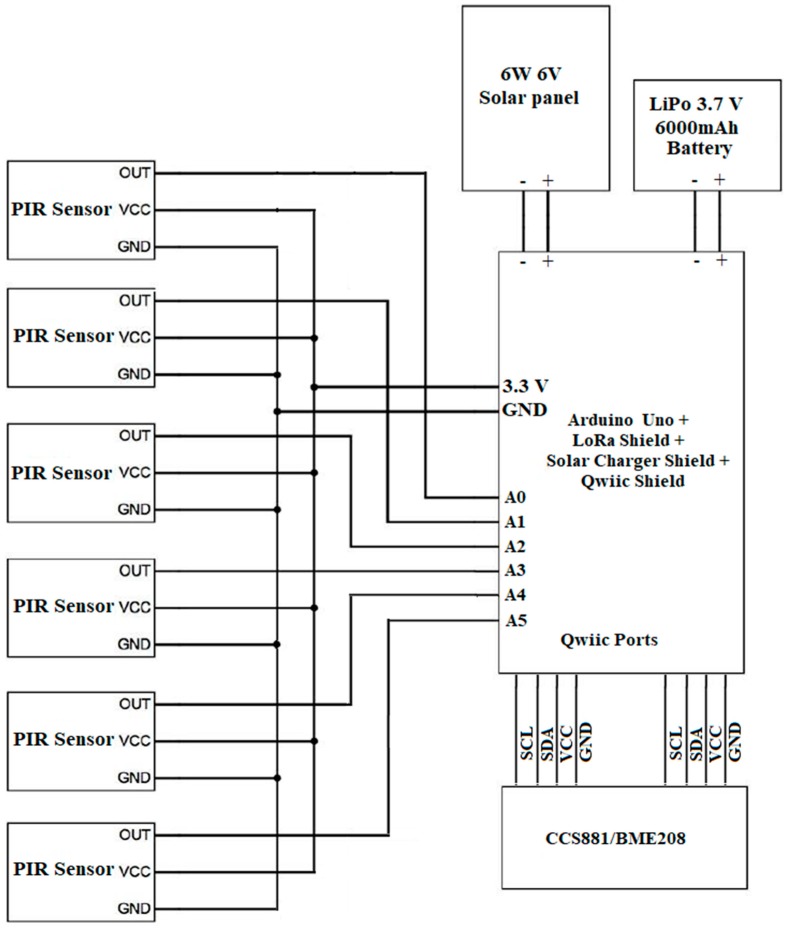
Circuit diagram of the proposed system.

The proposed system consists of six PIR sensors, one environmental combo sensor [39], QWIIC shield [40], Arduino UNO microcontroller [41], power converter [42], LoRa shield [43] for radio communication, solar panel [44] and rechargeable battery [45] to provide continuous energy. The PIR sensors are connected to the analog inputs of Arduino Uno. The environmental combo is connected to the Arduino Uno via the QWIIC shield that has four 3.3 V I^2^C ports and I^2^C sensors are connected to the shield via the QWIIC cable [46]. The solar charger shield and the LoRa shield are also connected with the main microcontroller. The 6000 mAh rechargeable battery and 6 V solar panel are connected with the power management block. A summary of the types of sensors and other electronics are listed in Table 3 and the circuit block diagram of the proposed system are shown in Figure 3.

**Figure 4:**
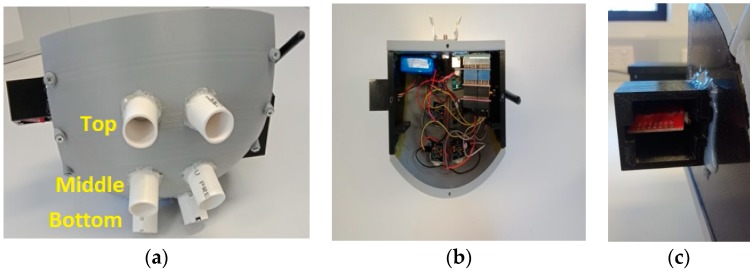
(**a**) Designed mould for accommodating the PIR sensors and other electronic devices and (**b**) internals of the system, (**c**) mould for the environmental combo sensor.

All the sensors and the necessary electronics were placed inside the mould that is designed using the Fusion 360 software, as shown in Figure 4a. A curvature plane was created to place the PIR sensors at the proposed angles. The PLA (PolyLactic Acid) filament was used to print the design in the DREAMER 3D PRINTER [48]. PIR sensors are placed in the holder in such a way that the E, D and C sections function for the bottom, middle and top pairs of the PIR sensors, respectively. Due to the usage of the holders, direct sunlight cannot affect the performances of the PIR sensors. A unique environmental combo sensor’s holder was designed to hold the sensor, which was connected with the glue on the box. One side of the holder is open so that it is exposed to the environment and able to read the environmental parameters such as temperature, humidity, pressure, CO_2_ and TVOC. The processing units and all the connections are located inside the mould.

### 2.4. Algorithm of the Pedestrian Count

To use the PIR sensors as the human counter, an intelligent algorithm has been developed to count accurately the number of pedestrians within the focus area of the PIR sensor. A flag variable and counter variable are used in this algorithm. Initially, the flag variable is stated at a LOW state. Usually, the output is LOW for the PIR sensor. The PIR sensor changes from LOW to HIGH state as soon as any person enters its focus zone and stays at the HIGH state for a certain duration before it goes back to LOW state. The time duration can be adjusted and is set in such a way so that the person takes a similar amount of time to walk the duration of its focus angle. Due to the change of state of the PIR sensor from LOW to HIGH, the value of the counter variable is increased by one and immediately the state of the flag variable is switched to HIGH state. This algorithm prevents the counter from counting more than once for the duration a pedestrian takes to cross the coverage region of the PIR sensor. As soon as the sensor goes LOW, the flag variables state is also changed to LOW and waits for the next person to cross.

**Figure 5:**
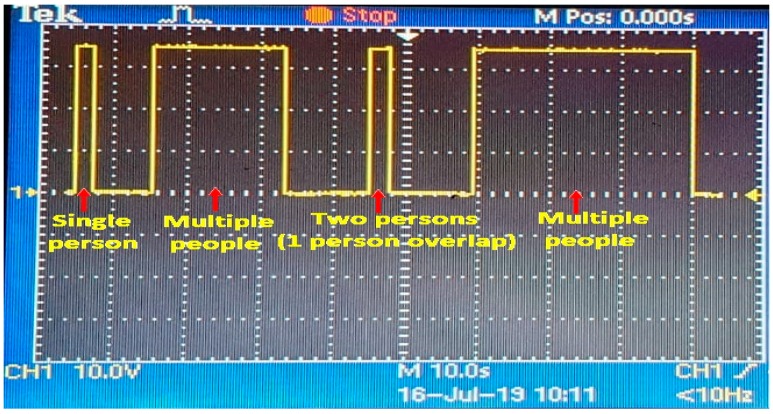
Change in duration of HIGH state of the passive infrared (PIR) sensor due to single and multiple passerby.

The direction of the pedestrian is determined from their entry time of the incident IR beam. The width of the footpath is divided into three zones, and each zone is covered with one pair of the PIR sensors. When a pedestrian is crossing a zone, he will trigger both the sensors in the pair. By recording the entry times of each sensor beam and comparing the earliest time among them, the direction is detected. This algorithm may provide incorrect results if a second person enters the focus zone of a PIR sensor before the transition from HIGH to LOW due to the first person. In this case, the duration of HIGH state gets extended as shown in Figure 5 and multiple passersby can be counted as a single individual. To avoid such a scenario, an error correction algorithm is developed.

The error correction algorithm is based on a comparison of the average time of crossing (ATC) a PIR sensor coverage area by a human, with the time duration at HIGH state (TDHS) of the corresponding sensor. The average time required to cross the coverage region (*d_p_*) of the PIR sensor by a human walking with a speed (*V_p_*) is found as:

(4) $$ATC=\frac{d_{p}}{V_{p}}$$

This algorithm mathematically calculates the number of the people, i.e., the increment of the counter variable as:

(5) $$N_{c}\approx round\;off\;\left(\frac{TDHS}{ATC}\right)$$

The error correction is activated for any PIR sensors that stay HIGH for more than 1.5 times of the ATC considering 50% tolerance. Before adding the corrected increment to the counter variable, it also compares the TDHS of the other sensor in the pair. Each of the paired sensors is placed in the mould at an angle that produces a 20 cm gap between their coverage regions. Therefore, the probable time period of crossing (PTPC) between the paired sensors is calculated as:

(6) $$PTPC=\frac{\begin{array}{c} \left(Coverage\;width\;of\;1st\;sensor\;in\;the\;Pair\right)\\ +\left(gap\right)+\left(Coverage\;width\;of\;2nd\;sensor\;in\;the\;same\;Pair\right) \end{array}}{Average\;walking\;speed\;of\;human}$$

If the values of TDHS for both the sensors in a pair are 75% near each other or the value of the counter variable is more than one for any PIR sensor in a pair within the PTPC, the increment is added. Otherwise, it does not add any increment correction. After counting the number of pedestrians by each pair of the PIR sensor, the total number of pedestrians is calculated as follows:

(7) $$N_{total}=\sum N_{c}$$

To find a more accurate value of the pedestrian count, the *N_total_* has been normalized using calibrated characteristics between the actual counted value and system counted value as explained in Section 3.1. Due to considering all the possible scenarios of the pedestrians walking pattern and feeding the system with actual count trends to ensure that the system provides more accurate results, the developed algorithm is defined as an intelligent algorithm.

**Figure 6:**
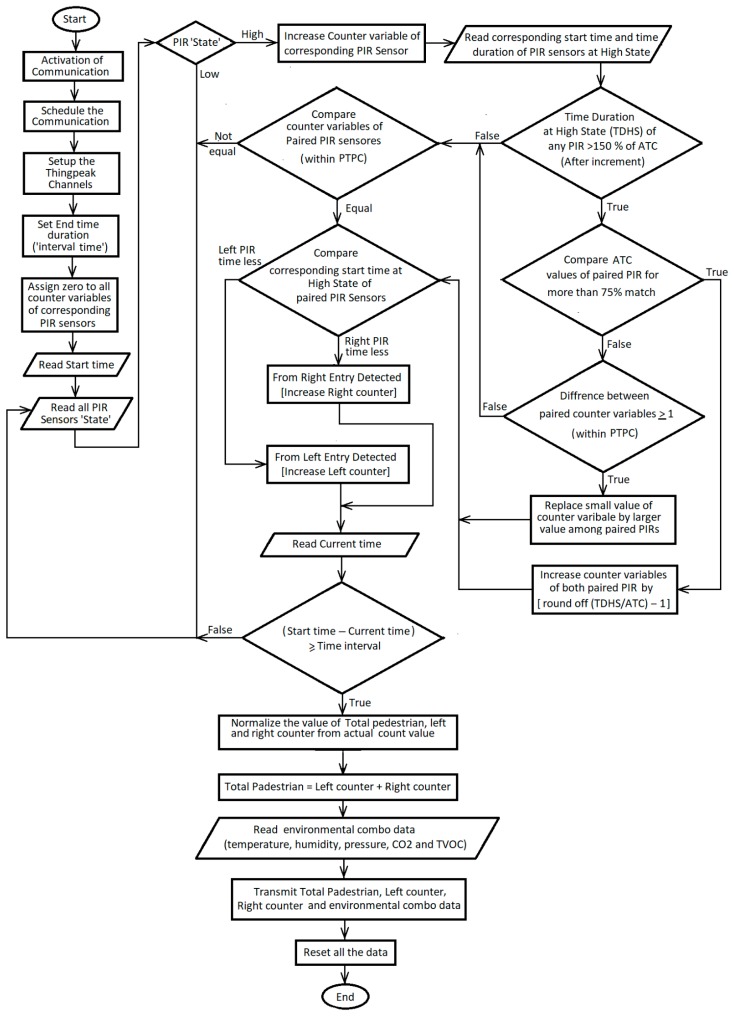
Flow chart of the pedestrian count, direction of travel and environmental monitoring system for a smart city.

Once the pedestrian counting and direction of travels are obtained for 10 min and environmental combo data are read. After reading all the sensor data, data is sent to the Thingspeak [49] server through the TTN (The Things Network) [50] gateway. The complete flow diagram of the developed system is shown in Figure 6.

### 2.5. Study Location

**Figure 7:**
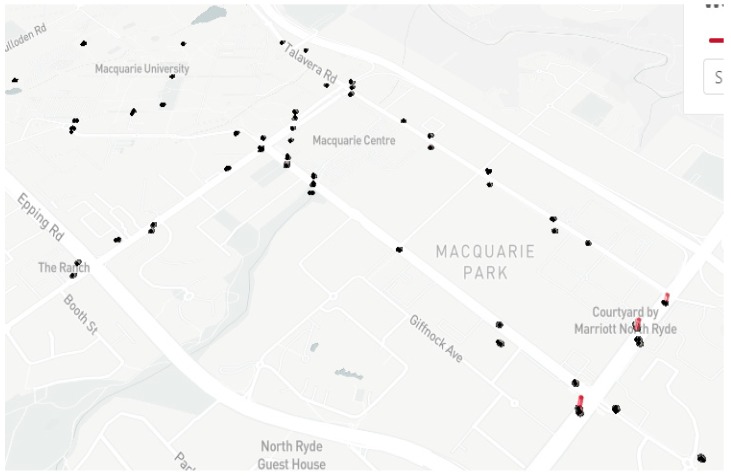
Study locations of the proposed sensor node.

**Figure 8:**
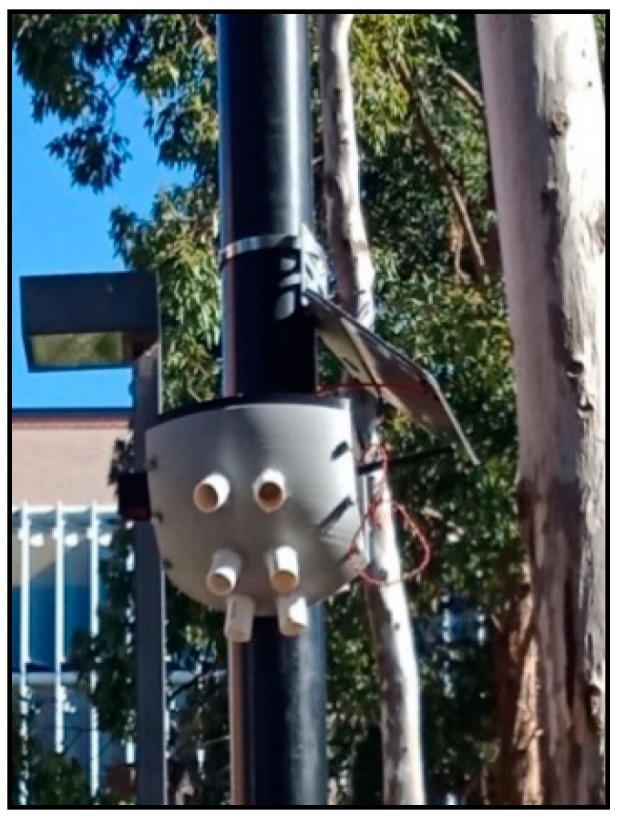
Proposed system installed on the electric pole.

There is a total of 74 sensors that have been installed near Macquarie University. Figure 7 shows the locations of all the sensor nodes. The sensor nodes have been working successfully for the last six months and providing the number of pedestrians, direction of travel and ambient parameters. Pole brackets were used to mount the proposed systems and solar panel on the electric pole. Figure 8 shows the mounting system with the solar panel on the pole.

### 2.6. Data Transmission and Collection

**Figure 9:**
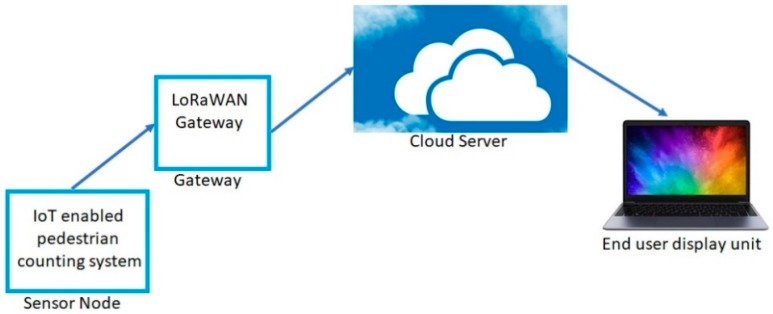
Block diagram of the Lora based pedestrian counting system.

The proposed pedestrian counting system is based on the Lora Communication. It offers connectivity of a few kilometers in urban areas and up to ten kilometers in rural areas. It is widely used where low data rate, low power and low throughput are required. Utilization of license-free Industrial, scientific and medical (*ISM*) radio bands enables LoRaWAN to attract customers such as the smart city [51]. This communication system consists of two different layers. The physical layer is based on the Chirp Spread Spectrum (CSS) modulation technique and the MAC-layer protocol is accountable for getting access to the network architecture [52]. The frequency shift keying (FSK) is used in the LoRa communication to communicate between the gateway and sensor node [53]. Figure 9 shows the block diagram of the proposed system. After the installation of the sensor nodes, the data were collected and stored in the Thingspeak channel.

## 3. Results and Discussions

This section describes the performance of the proposed system by comparing the system count with the manual count. It also explains how the accuracy of the system is improved. This section shows how collected data are displayed on the Thingspeak channel.

### 3.1. Data Collection and Validation

The developed system was installed on the pole at Macquarie University. The data was collected from the proposed system. The number of pedestrians, pedestrians coming from the left side and right side were also counted manually. The data was counted manually from 8:00 a.m. to 10:00 p.m. The flow of pedestrians changed due to the peak and off-peak time. The counting was done for three days (weekdays) continuously, and the average data were taken for data analysis. Table 4 shows the data of the manual count from 8:00 a.m.–10:00 p.m. with a 1-h time interval. A marker is used at 1 m height of the pole to count the animals in two different height groups such as below 1 m or above 1 m. The system is not capable of distinguishing among humans and pets taller that one meter. While doing the manual counting, no pet is found having a height more than 1 m. It can be said from this scenario that urban people prefer keeping smaller pets than taller pets. This nullifies the probability of error counting animals as humans by the system. The maximum error that occurs in this system counting is less than 10%.

**Figure 10:**
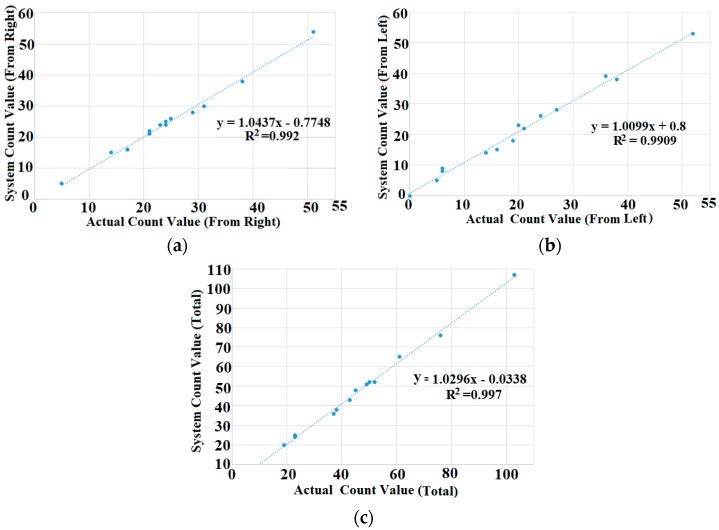
Comparison between the actual count and system count. (**a**) Pedestrian coming from the right, (**b**) pedestrian coming from the left and (**c**) total number of pedestrians.

In order to improve the accuracy, the manual count and system count data are plotted against each other, and the equation for determining the actual count is obtained. Figure 10a–c shows the relationship between the system count and the actual count for right, left and total pedestrians that follow the linear equations with a coefficient of determination (R^2^ = 0.992, 0.9909 and 0.997) from the actual data. This linear equation was used to calculate the actual count from the system’s count, which can be represented as follows:

Pedestrians coming from the right side,

(8) $$x=\frac{y+0.7748}{1.0437}$$

Pedestrians coming from the left side,

(9) $$x=\frac{y-0.8}{1.0099}$$

Total number of pedestrians,

(10) $$x=\frac{y+0.0338}{1.0296}$$

where x represents the actual pedestrian count and y represents the system’s count. These equations were used as fitted lines for the final system.

After doing the corrections, the system was installed again on the pole and data was collected manually. Both data were compared. Table 5 shows the comparison between the corrected results versus the actual results. As no animal is found having height more than 1 m, the number of animals crossing the coverage region of the sensor node is not included in Table 5. It is seen from the table that the developed system’s accuracy is improved to 95% after the correction.

The developed system provides highly accurate data without affecting human privacy compared to a camera-based system. An environmental combo sensor is also included in the system that provides temperature, humidity, pressure, CO_2_ and TVOC. Data collected from the sensor are compared with the environmental data from the Sydney weather station [54]. The sensors were calibrated by calculating the offset factors and applying to the BME280/CCS811 data. There are 74 nodes that are developed and installed close to Macquarie University around Herring Road (33°46′43.4″ S 151°06′59.8″ E), Waterloo Road (33°46′44.8″ S 151°07′13.3″ E), and Talavera Road (33°46′35.1″ S 151°07′22.7″ E).

### 3.2. Data Transfer to IoT Cloud Server

**Figure 11:**
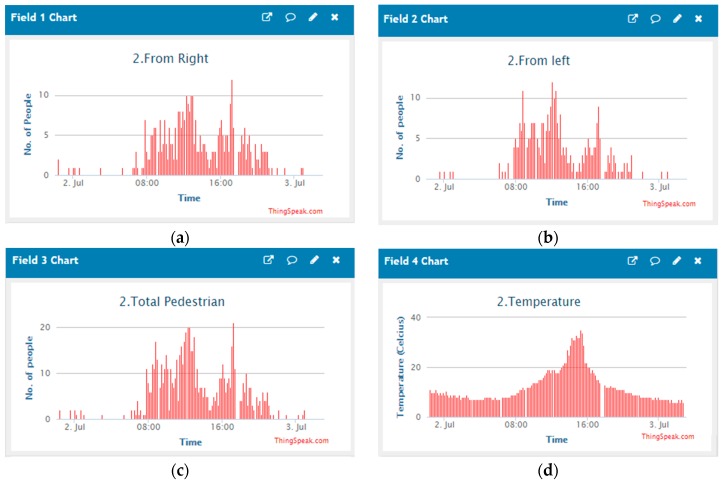

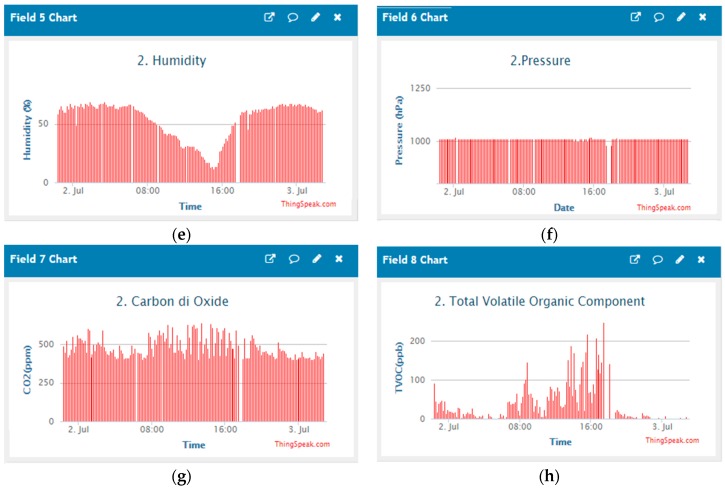
Transferred (**a**) pedestrians coming from the right, (**b**) pedestrians coming from the left, (**c**) total number of pedestrians, (**d**) temperature (°C), (**e**) humidity (%), (**f**) pressure (hPa), (**g**) carbon di oxide (ppm) and (**h**) total volatile organic component (ppb) data from the system to the Thingspeak cloud server.

A Thingspeak cloud server is used to store the data which is a free IoT cloud server for data storage. Sensor data were transmitted to the server every 10 min for increasing the battery time. The sampling time is controlled through Arduino programming. Figure 11 shows the real-time data of the developed system for few data. A Thingspeak channel can support a maximum of eight fields and various fields were reserved for the pedestrian coming from the right, left and total, temperature, humidity, pressure, CO_2_ and TVOC. All the data were collected through the TTN gateway.

From Figure 11 the variation of the pedestrian numbers and direction of travel can be seen, and the pattern of the pedestrian flow is understood. A maximum number of pedestrians are walking during a particular time such as 08:00–10:00, 12:00–14:00, and 14:00–18:00 due to the starting of office time, lunchtime and ending of office time. Identical data can be seen for all three days. During the night, a minimal number of people can be seen.

### 3.3. Power Consumption

One of the significant outstanding challenges of the wireless sensor network (WSN) system is the selection of the appropriate sensor and communication technology since battery scaling is the main limit to sensor nodes miniaturization. The replacement of the battery is not possible or feasible in many cases. Therefore, efficient energy management is essential. The simplest way to reduce power consumption is to use low-power devices and passive sensors (such as the PIR sensors). A low-power microcontroller such as Arduino Uno has only 32 Kbytes of Flash memory and 2 Kbytes of SRAM. Power consumption in wireless sensor nodes has peaked when the radio is active. Therefore, wireless communication should be limited. Power consumption by the sensor node can also be limited by increasing the idle time and reducing the active time. Table 6 shows the current drawn by the sensors node in 10 min, i.e., 1 cycle. The current drawn by each and every component is calculated by measuring the amplitude of the voltage waveform from the oscilloscope across a 0.5 Ω resistance connected in a series with the microcontroller. Total power consumption by the sensor node including transmission is given by,

(11) $$P_{s}=V_{s}\times I_{n}+V_{s}\times I_{td}\times t_{td}\times f_{td}$$

where,

*V_s_* = nominal voltage of the sensor node = 3.3 V
*I_n_* = current drawn by the sensor node in 1 h = 40.01 mA
*I_td_* = current drawn during transmission = 120 mA
*t_td_* = time duration of data transmission = 60 mc
*f_td_* = frequency of data transmission = $\frac{1}{10\;\mathrm{mins}}=\frac{1}{10\times 60}=\frac{1}{600}\;\mathrm{Hz}$

The current drawn by the sensor node, *I_n_* is calculated based on the idle time and active time of the electronic devices in 10 min, i.e., 1 cycle.

Therefore, the total current consumption by the sensor node,

$$I_{n}=\frac{12,000+10,800+599+13+599.94}{10\times 60}\Rightarrow I_{n}=40.01\;\mathrm{mA}$$

Power consumption, *P_s_* is found as

$$\Rightarrow P_{s}=\left(3.3\times 40.01+3.3\times 120\times \frac{60}{10^{3}}\times \frac{1}{600}\right)\;VA\Rightarrow P_{s}=132.07\;\mathrm{mVA}$$

Assuming the efficiency of the power converter is 90%, the minimum amount of the required mVA from the battery, $P_{bs}=\frac{132.07}{0.90}=146.74\;\mathrm{mVA}$.

Given the battery voltage *V_B_* = 3.7 V, the discharge current required from the battery is,

$$I_{dis}=\frac{146.74}{3.7}=39.66\;\mathrm{mA}$$

Therefore, the lifespan of the 3.7 V 6000 mAh battery can be calculated as follows:

$$B_{Life}=\frac{6000}{39.66}=151.28\;\mathrm{hrs}$$

It is assumed that the battery will be able to discharge 100%, though it is not able to do so in the practical scenario. From the above numerical calculations, it is found that the transmission of data every 10 min enables the battery sustainability to 151 h. The battery is charged by the 6 V 6 W solar panel during daylight. There will be continuous power to the system unless the sun does not come out at all for a consecutive six days.

## 4. Conclusions

An IoT enabled pedestrian count, direction of travel and ambient parameters determination system is successfully presented in this paper. The PIR sensors detect the movement and convert the data into the pedestrian count and direction of movement. The developed system is tested at various times of the day to evaluate the performance. This novel method counts pedestrians with 95% accuracy and avoids counting any movement of domestic animals such as dogs or cats. The method does not require any images or videos thus protecting human privacy. The environmental combo provides all the necessary information of the weather such as temperature, humidity, pressure, CO_2_ and VOC. The installation of the sensor nodes on the electric pole reduces the financial burden. The implementation cost of the system is about 250 US dollars, which includes the purchase of electronic consumables in small numbers. The total cost of fabrication will significantly reduce if the productions are made in large numbers. The current price though is less compared to other existing systems thus making this a potential candidate to implement in a smart city.

The open-access database of the system promotes identifying hotspots and taking necessary action for pedestrian safety and facilities improvement. Moreover, it will help the City Council to improve the planning and provision of transportation services. Furthermore, this will enable local businesses to monitor the pedestrian traffic flow and manage business resources to improve efficiency and increase revenue. Environmental monitoring data will help in understanding air quality and taking essential measures for making the city greener. Therefore, the existence of such sensor nodes in a city provides an opportunity for decision-makers to test assumptions and scenarios, reducing the chance of costly mistakes with the infrastructure. That is why it is adopted by the Macquarie park district, Sydney, Australia, which is experiencing considerable growth of office developments over the past two decades and is expected to continuingly expand as Sydney’s knowledge hub in near future. Without such a mutual policy and implementation framework, urban planning projects may place tremendous pressure on the precinct, transportation and inhabitants whilst also leading to costly and ineffective solutions. It is anticipated that the major cities in Australia and other countries will adopt this technology very soon. In addition to pedestrian counting, this system can also be implemented in a smart building to know the number of people inside and outside the building during emergency evacuation.

Although there are so many advantages of the proposed system, there are some limitations as well. This system is not able to differentiate between humans and animals having height more than 1 m. Due to keeping 1 m clearance from the street level, babies sleeping on the pram are not counted.

Future work would be the inclusion of more sensors such as noise sensor, wind speed determination, rain sensors, visibility sensor, etc. Moreover, the long term collected data would be analyzed to predict the trends. The environmental effect on the pedestrian movement would also be analyzed for different applications.

## Figures and Tables

**Table 1:** Comparison of the existing pedestrian counting system in smart cities worldwide.

City	Sensing Tech.	Features	Installation	Comm. Tech.	Power Consumption	Cost	Ref
Melbourne, Australia	A thermal and laser-based sensor	Pedestrian count	Separate installation	3G	High	High	[31]
Liverpool, Sydney, Australia	Video camera	Pedestrian and vehicle count	On electric pole	LoRa	Low	High	[32]
Auckland, New Zealand	Thermal camera	Pedestrian count and direction of travel	Under the canopy or on electric pole	3G	High	High	[33]
New York, NY, USA	Video camera	Pedestrian count	electric pole	4G	High	High	[34]
Dublin, Ireland	Video camera	Pedestrian count and direction of travel	Separate installation	3G	High	High	[35]
Barcelona, Spain	Thermal camera	Pedestrian count	Separate installation	3G	High	High	[36]
Macquarie University, Australia. (This system)	PIR sensor	Pedestrian count, direction of travel, temperature, humidity, pressure, CO_2_ and TVOC	on electric pole	Lora	Low	Low	[This Work]

**Table 2:** Measured coverage ranges of different regions of Fresnel lens at full and reduced sensitivity mode.

Name of the Section of Fresnel Lens	Maximum Coverage Length at Full Sensitivity (m)	Minimum Coverage Length at Reduced Sensitivity (m)
C	4	1.8
F	5	2.25
E	6.6	3
D	7.2	3.25
B	7.6	3.5
A	10	4.5

**Table 3:** Summary of the electronics used in the proposed system.

Name of the Item	Description
Arduino Uno Rev 3	Development board
Parallax Inc. 555-28027 PIR sensor module	Motion detection sensor
CCS811/BME280 (Qwiic) Environmental Combo	CCS811 provides equivalent CO_2_ (or eCO_2_) in the parts per million (PPM) and total volatile organic compounds in the parts per billion (PPB). BME280 provides humidity (%), temperature (°C), and barometric pressure (Pa).
Qwiic Shield	Incorporate Environmental Combo with Arduino
Seed Studies Solar charger shield v2.2	Power management block
6V 6W solar panel	Solar panel from Voltaic
LoRa Shield for Arduino 915 MHz	Long-range transceiver
Polymer Lithium Ion Battery 3.7 V 6000 mAh	Rechargeable battery
ANT-916-CW-HWR-SMA [47]	External antenna

**Table 4:** Actual and system pedestrian count in the timeline.

Time	Actual Count					System Count		
	Animal		Pedestrian			Pedestrian		
	Bellow 1 m	Above 1 m	Total	Right	Left	Total	Right	Left
08:00–09:00	0	0	61	25	36	65	26	39
09:00–10:00	0	0	50	23	27	52	24	28
10:00–11:00	0	0	45	21	24	48	22	26
11:00–12:00	1	0	76	38	38	76	38	38
12:00–13:00	0	0	103	51	52	107	54	53
13:00–14:00	0	0	43	24	19	43	25	18
14:00–15:00	0	0	23	17	6	24	16	8
15:00–16:00	5	0	37	21	16	36	21	15
16:00–17:00	3	0	49	29	20	51	28	23
17:00–18:00	0	0	52	31	21	52	30	22
18:00–19:00	2	0	38	24	14	38	24	14
19:00–20:00	0	0	19	14	5	20	15	5
20:00–21:00	0	0	23	17	6	25	16	9
21:00–22:00	0	0	5	5	0	5	5	0

**Table 5:** Validation of the result between the developed system and manual counting.

Time	Developed System	Manual Counting	Error (%)
10:00–11:00	97	102	5
11:00–12:00	73	76	4
12:00–13:00	103	107	4
13:00–14:00	44	46	4
14:00–15:00	54	56	4
15:00–16:00	42	44	5
16:00–17:00	79	81	2
17:00–18:00	49	51	4
18:00–19:00	29	30	3
19:00–20:00	35	36	3

**Table 6:** Measured current drawn by the electronic devices of the sensor node in one cycle.

Name of the Component	Time	Current Consumption (mA)	Current Drawn in One Cycle (mAsec)
Arduino Uno MCU	10 min	20	20×10×60=12,000
six PIR sensors	10 min	18	18×10×60=10,800
Env. combo (idle)	9 min 59 s	1	1×(9×60+59)=599
Env. combo (active)	1 s	13	1×13=13
LoRa shield(idle)	9 min 59 s 940 ms	1	$1\times \left(9\times 60+59+\frac{940}{1000}\right)=599.94$
